# Photon counting CT vs. flat-panel CT in the evaluation of enhancement patterns in chronic subdural hematoma after middle meningeal artery embolization

**DOI:** 10.3389/fneur.2025.1608308

**Published:** 2025-05-16

**Authors:** Christoph J. Maurer, Lars Behrens, Stefan Schiele, Mahmoud Zaki, Guilherme Quint, Christina Wolfert, Björn Sommer, Franz J. Stangl, Ansgar Berlis

**Affiliations:** ^1^Diagnostic and Interventional Neuroradiology, Faculty of Medicine, University of Augsburg, Augsburg, Germany; ^2^Computational Statistics and Data Analysis, Institute of Mathematics, University of Augsburg, Augsburg, Germany; ^3^Neurosurgery, Faculty of Medicine, University of Augsburg, Augsburg, Germany

**Keywords:** photon counting CT, middle meningeal artery embolization, chronic subdural hematoma, contrast enhancement, hemorrhage differentiation

## Abstract

**Introduction:**

Middle meningeal artery embolization is a treatment option for chronic subdural hematoma (cSDH), but data on post-procedural imaging interpretation remain limited. This study investigates whether specific enhancement patterns can predict radiological outcomes and evaluates the utility of photon-counting computed tomography (PCCT) in distinguishing contrast enhancement from hemorrhage.

**Methods:**

We retrospectively analyzed 105 cSDHs imaged with either PCCT or flat-panel CT immediately after embolization. Two independent raters assessed enhancement patterns; diagnostic confidence and interrater agreement were evaluated.

**Results:**

PCCT demonstrated higher diagnostic confidence and interrater reliability than flat-panel CT. Internal enhancement and fluid–fluid levels were significantly associated with hematoma persistence or recurrence.

**Discussion:**

PCCT enhances post-embolization imaging assessment in cSDH. Specific enhancement patterns may serve as imaging biomarkers to identify patients at increased risk for unfavorable radiological outcomes.

## Introduction

Chronic subdural hematomas (cSDH) have been extensively studied since their first scientific report in 1675 by Johann Jakob Wepfer (1). Rudolf Virchow was the first to describe in detail the chronic inflammation and thickening of the dural layers associated with neoangiogenesis and to recognize these fragile, newly formed vessels as the source of recurrent bleeding into the hematoma (2). The basic concept remains the same today and underpins our understanding of the formation and persistence of cSDHs (3–5). However, these pathogenesis findings, have not yet provided an effective therapeutic target. Although various medical treatments have been tested unsuccessfully, discontinuation of anticoagulants and surgical evacuation with burr hole irrigation remain the recommended treatment for these patients (6–8).

In recent years, embolization of the middle meningeal artery (MMA), either as a sole or adjunctive treatment for cSDH using particles or liquid embolic agents, has received considerable attention and randomized controlled trials have been conducted to determine the benefit of embolization (9–16). The recently published results of three large randomized controlled trials—EMBOLIZE, MAGIC-MT, and STEM (17–19)—demonstrate that MMA embolization significantly reduces the risk of treatment failure in patients with nonacute subdural hematoma compared to conventional management, without an associated increase in severe perioperative adverse events; however, definitive benefit across specific patient subgroups remains to be established (20).

A common finding after embolization are the enhancement patterns of various parts of the dura and the subdural hematoma itself due to the injection of contrast media and embolic agents, especially particles, in the middle meningeal artery (21–23). These enhancement patterns appear to be related to the pathophysiology of subdural hematomas and may correlate with neovascular membranes and pseudomembranes. They can lead, via leaky capillaries, to the extravasation of contrast media into the subdural space itself. However, the significance of these enhancement patterns and their potential prognostic value remain unclear. Additionally, distinguishing between post-therapeutic enhancement and new hemorrhage on conventional or post-interventional flat-panel CT, based solely on Hounsfield units (HU), can be challenging and seems to be more effective with Dual-Energy CT imaging (21). Photon counting CT (PCCT) imaging can also reliably differentiate and quantify, contrast enhancement, hemorrhage, and even distinct embolic agents (24, 25); however, it has not yet been tested specifically in the context of MMA embolization for cSDH.

Therefore, the aims of our study were twofold: first, to compare flat-panel CT and PCCT in the detection and characterization of enhancement patterns in cSDH after MMA embolization, with particular emphasis on PCCT’s ability to distinguish enhancement from hemorrhage; second, to evaluate whether specific enhancement patterns are predictive of short- and long-term outcomes, including hematoma persistence or recurrence with or without surgical evacuation.

## Materials and methods

### Study design

This single-center, retrospective, observational study was approved by the Institutional Review Board of the Ludwig-Maximilian-University of Munich. It was conducted in accordance with the ethical standards of the Declaration of Helsinki (1964) and its subsequent amendments. Due to the study’s retrospective nature and the irreversible anonymization of patient data, individual informed consent was not required.

### Patients

All consecutive patients treated with MMA embolization for cSDH between January 2020 and November 2022, who underwent either PCCT (NAEOTOM Alpha; Siemens Healthcare, Erlangen, Germany) or flat-panel CT (Axiom Artis; Siemens Healthcare, Forchheim, Germany) immediately after the procedure. Clinical data were collected retrospectively from our electronic databases. Medical records were assessed for several patient parameters including sex, age, hematoma size at presentation, and all available follow-up images. The material used, the interventional procedure, and the peri- as well as postprocedural complications were documented. In case of insufficient quality of the CT images, patients were excluded from the analysis.

### Image reconstruction and analysis

Flat-panel CT data were transferred to an independent workstation (Leonardo, Siemens Healthcare, Germany) to generate reformatted images in three standard planes (transverse, coronal, and sagittal). The PCCT datasets were iteratively reconstructed on the scanner console, preserving the spectral image information for further analysis. Dedicated image analysis was performed on an independent workstation (syngo.via, version VB80C; Siemens Healthcare, Erlangen, Germany) in a dual-energy workflow (virtual unenhanced application profile). Conventional images (CI), iodine maps (IM), and virtual noncontrast (VNC) series were reconstructed.

### Image evaluation

**Figure 1:**
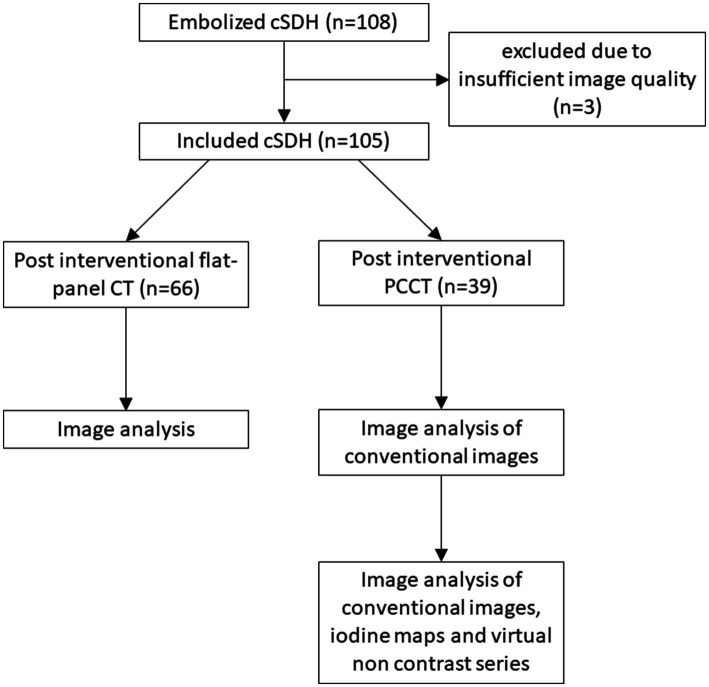
Flowchart illustrating the image evaluation.

In cases of bilateral cSDHs, each hematoma was treated independently and assessed by two raters (LB and CJM, both 19 years of radiological and neuroradiological experience). The raters were blinded to follow-up images and the clinical course. However, the raters were not blinded to the imaging modality due to the inherent distinct imaging characteristics of the two modalities. Post-interventional images were presented alongside previous radiological images to replicate the clinical setting following MMA embolization. For PCCT scans, the conventional images were analyzed first. In a subsequent step, all available information, including CI, IM, and VNC images, were provided for analysis and rated independently from the initial step. The study algorithm is shown in Figure 1.

**Figure 2:**
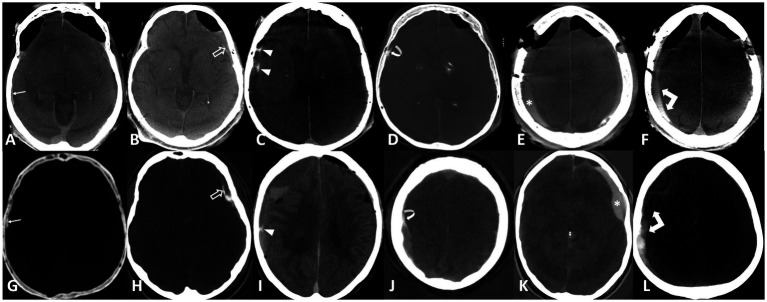
Enhancement patterns: Upper row flat-panel CT, lower row iodine maps of photon counting CT. **(A,G)** Outer membrane (arrows), **(B,H)** inner membrane (open arrows), **(C,I)** septations (arrowheads), **(D,J)** spandrel sign (curved arrows), **(E,K)** internal enhancement (asterisk), **(F,L)** fluid–fluid level (double arrows).

The raters evaluated the diagnostic quality of the CT images as either sufficient or insufficient, particularly due to the presence of motion artifacts. Images deemed to have sufficient quality were analyzed for the presence of hyperdensity within or around the hematoma. The raters further determined whether the hyperdensity represented hemorrhage, enhancement, or both, and rated their diagnostic confidence on a 5-point Likert scale (1 being very unsure to 5 being very sure). In cases of presumed enhancement, the raters identified all enhancing components, including the outer membrane, inner membrane, transition zone (‘spandrel sign’), septations, internal enhancement within the subdural hematoma, and fluid–fluid levels, as previously described (21). Examples of enhancement patterns are provided in Figure 2.

The final evaluation, including classification of hemorrhage and enhancement patterns, was based on the consensus of both readers after comprehensive review of all follow-up imaging and clinical data, and served as the reference standard for assessing the predictive value of these patterns with respect to hematoma recurrence, persistence, and the need for surgical intervention.

### Outcome

An unfavorable outcome on imaging was defined as either (1) persistent hematoma, meaning no measurable reduction in hematoma volume compared to the baseline pre-embolization scan; (2) progressive hematoma, defined as an increase in volume; or (3) the need for additional surgical interventions such as burr hole drainage or craniotomy. Short-term outcome was assessed within 6 weeks post-embolization, while long-term outcome was determined based on the most recent available clinical and imaging follow-up.

### Statistical analysis

Descriptive statistics were employed, with the median and interquartile range (IQR) reported for continuous and ordinal variables. Interrater variability was assessed using Gwet’s AC1 to evaluate agreement among raters. Gwet’s AC1 values can range from −1 to 1, where values closer to 1 indicate stronger agreement between raters, with common benchmarks being: almost perfect (0.81–1.00), substantial (0.61–0.80), moderate (0.41–0.60), fair (0.21–0.40), slight (0.01–0.20), and poor (≤0.00). Gwet’s AC1 was chosen since it is preferred for imbalanced distributions. Subjective assessment of confidence was compared between PCCT and flat-panel CT using the Wilcoxon rank-sum test for each individual rater. Outcome analysis for each pattern was conducted using univariate Bayesian logistic regression to address the low number of events and to facilitate calculation despite separation. A Student t distribution (df = 7, location = 0, scale = 2.5) was used as a non-informative, symmetric prior around zero for the intercept and log odds of the variable. The model simulation was conducted with a Monte Carlo Markov Chain (MCMC) algorithm from the rstanarm package in R. Odds Ratios and 95% Bayesian credible intervals based on quantiles were reported. A variable was considered significant if the 95% credible interval does not contain 1. The statistical analyses were conducted using R (Version 4.3.1).

## Results

### Patients’ characteristics

A total of 105 cSDHs were included in the final analysis. Three additional cases were excluded prior to analysis due to insufficient image quality, predominantly caused by motion artifacts. All patients underwent embolization of the MMA. Of the treated 105 cSDHs, 20 (19%) were located on the right side, 30 (29%) on the left side and 55 bilateral (52%). Laterality was not significantly associated with unfavorable outcomes. In the majority of cases, middle meningeal artery embolization was performed postoperatively following surgical evacuation of the cSDH; however, in 11 patients (10.5%), embolization was performed as a standalone primary treatment. In 23 patients (21.9%), only PVA particles were used; in 71 patients (67.6%), both PVA particles and coils for occlusion of the main trunk were used; and in 4 patients (3.8%), only coiling was performed. Liquid embolics were utilized in 7 patients (6.7%). Mean hematoma width was 17 mm (SD = 7). Three complications of the embolization procedure (3%) were documented: one patient with transient double vision, one patient with a TGA and one patient with an asymptomatic cerebellar infarction after embolization. All complications were asymptomatic or resolved within 24 h. The postinterventional CT was performed on average 26 min (SD = 32) after the final angiographic run. Sixty-six (63%) patients received a flat-panel CT after intervention, while 39 (37%) underwent PCCT. However, the time interval between embolization and imaging was significantly longer for PCCT (median 46.0 min, IQR 31.0–64.0) compared to flat-panel CT (median 2.0 min, IQR 2.0–4.5), attributable to the need for patient transport to the CT suite. Patients’ characteristics are detailed in Table 1.

**Table 1:** Patients’ characteristics.

Variable	Value
cSDHs	*n* = 105
Age Mean, SD (IQR)	77, 10 (73, 85)
Sex, *n* (%)	
Male	84 (80%)
Female	21 (20%)
Initial Hematoma width in mm	
Mean (SD)	17 (7)
Median	16
IQR	12–20

IQR, interquartile range; SD, standard deviation.

Short-term follow-up imaging within 6 weeks was available for 72 cSDHs, and long-term follow-up for 94 cSDHs. Four patients died due to causes unrelated to the cSDH (COVID-19 infection, prostate carcinoma, and cardiovascular events in two patients), and 7 cSDHs were lost to follow-up. The overall median duration of follow-up was 96 days (IQR 29–553 days). Recurrence was observed in 5 cSDHs (6.9%) on short-term follow-up and in 1 cSDH on long-term follow-up. Persistent cSDH was considered an unfavorable outcome and occurred in 7 cases (9.7%) on short-term and 7 cases (7.4%) on long-term follow-up. In the subgroup of patients with unfavorable long-term outcomes (persistent or progressive cSDH), embolization techniques were distributed as follows: 3 cases with PVA particles only, 5 cases with PVA particles and coils, and no cases with liquid embolics. No clear association between embolization technique and treatment failure could be identified due to the small sample size. In the subgroup of 11 patients treated with embolization alone, no cases of hematoma recurrence were observed; however, 3 cases showed no measurable hematoma resorption during long-term follow-up. Detailed follow-up imaging findings are summarized in Table 2.

**Table 2:** Imaging outcome.

Parameter	Short-term F/U	Long-term F/U
cSDHs, *n* (%)	72 (69%)	94 (90%)
Hematoma width in mm		
Mean	8.6	3.6
Median	8.0	0.0
IQR	5–13	0.0–6.8
Difference hematoma width in mm		
Mean	7.8	13
Median	8,5	13
IQR	3–13	9–18
Outcome, *n* (%)		
Progressive	5 (6.9%)	1 (1.1%)
Unchanged	7 (9.7%)	7 (7.4%)
Residual or complete resolution	60 (83%)	86 (91%)

### Diagnostic confidence and interrater agreement

**Figure 3:**
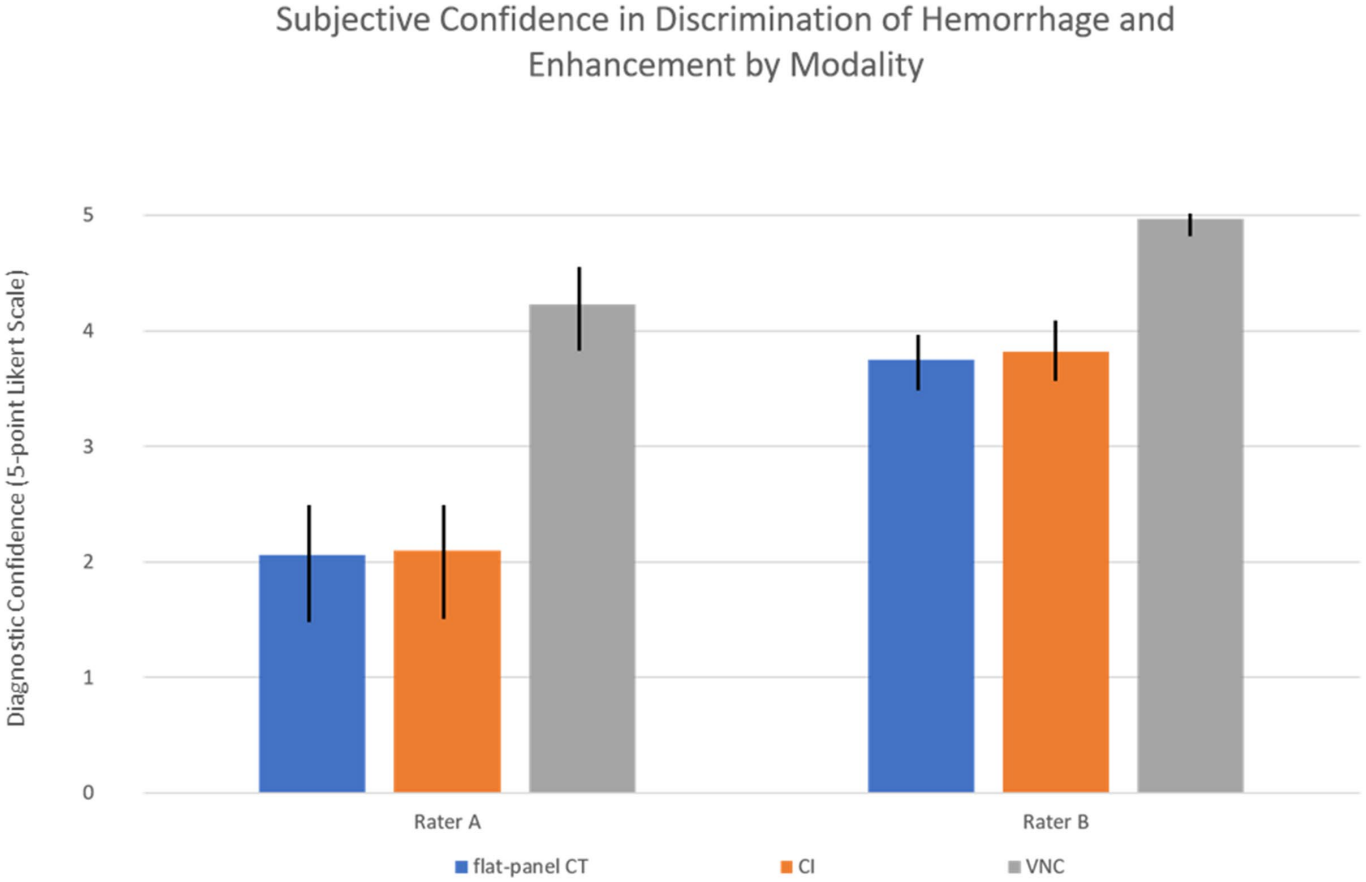
Rater confidence with standard deviation by modality. CI, conventional images; VNC, virtual non-contrast images. For both raters, the difference in confidence between modalities was statistically significant (*p* < 0.001).

The subjective assessment of confidence in discrimination between hemorrhage and contrast enhancement was for each rater significantly higher on PCCT (Rater A: 4.23, Rater B: 4.97) compared to CI (Rater A: 2.10, Rater B: 3.82) and flat-panel CT datasets (Rater A: 2.06, Rater B: 3.75), with a *p*-value < 0.001 for each rater. Differences in confidence by modality are shown in Figure 3.

Consistency between both raters was substantial for PCCT (Gwet’s AC1: 0.80) with agreement in 86.1% of cases, compared to moderate concordance for CI (Gwet’s AC1: 0.50) with agreement in 69.2% of cases and poor concordance for flat-panel CT (Gwet’s AC1: −0.25) with agreement in only 36.1% of cSDHs.

### Final consensus imaging evaluation

Hyperdense structures in cSDHs were diagnosed as consensus in 98 hematomas (93%). The assessment revealed hemorrhage alone in 1 cSDH (1.0%), contrast enhancement in 55 (56%) and both, hemorrhage and enhancement in 42 cSDHs (43%). Enhancement patterns are provided in Table 3.

**Table 3:** Enhancement patterns.

Enhancement patterns [consensus, *n* (%)]	Yes	No
External membrane	78 (74%)	27 (26%)
Internal membrane	44 (42%)	61 (58%)
Septations	7 (6.7%)	98 (93%)
Spandrel sign	34 (32%)	71 (68%)
Internal enhancement	59 (56%)	46 (44%)
Fluid–fluid level	44 (42%)	61 (58%)

**Figure 4:**
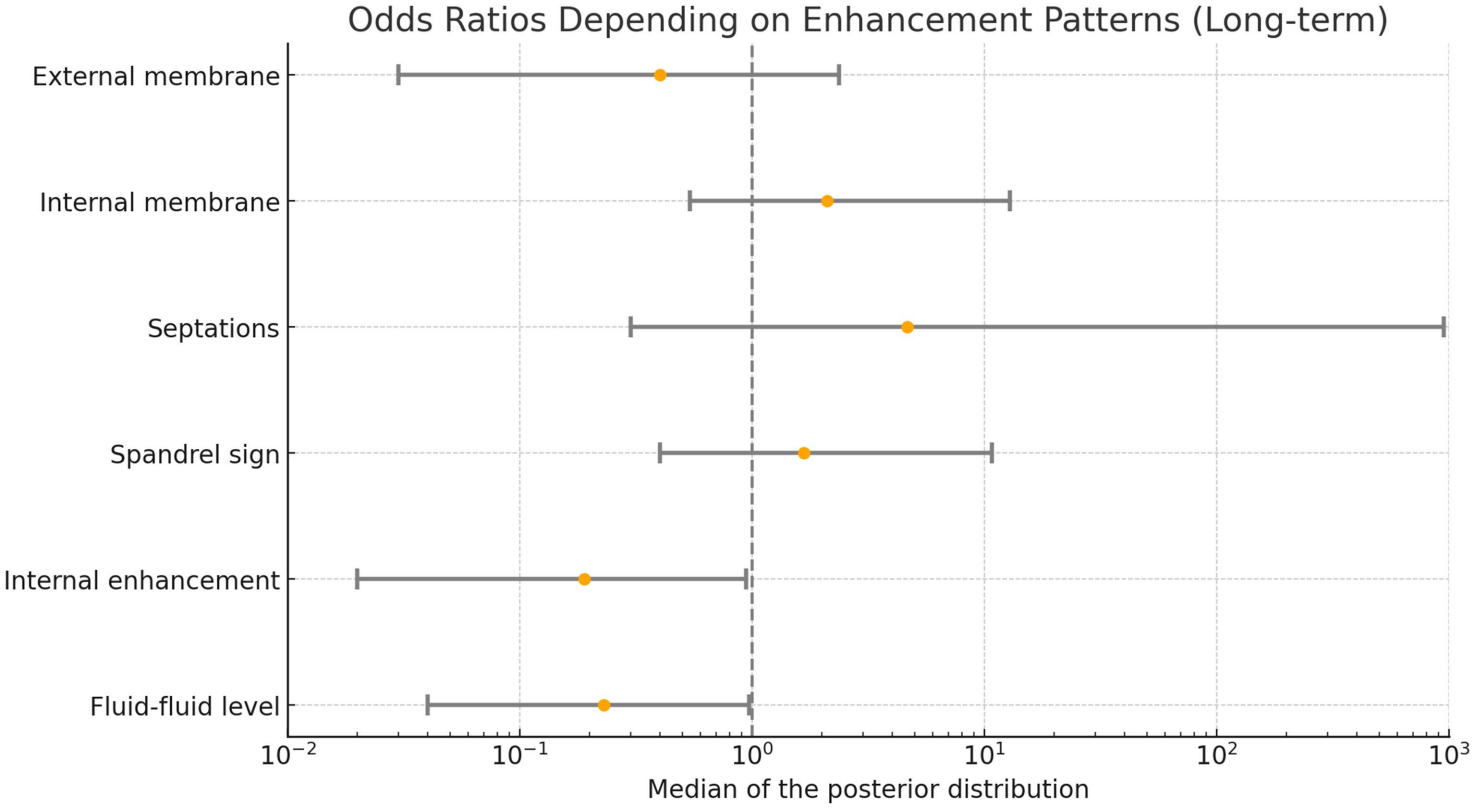
Odds ratios based on enhancement patterns. Values below 1 indicate an increased likelihood of hematoma progression or failure to decrease in volume (persistence).

The Bayesian logistic regression demonstrated an increased risk for an unfavorable outcome (persistence or progressive cSDH) for cSDH with contrast enhancement within the hematoma and fluid–fluid levels, as the 95% credible intervals for these variables did not include an odds ratio of 1, indicating statistical significance. Results of the regression analysis are provided in Figure 4 (long-term outcome).

## Discussion

Our data show that PCCT provides a significantly higher diagnostic confidence in discrimination of hyperdense hemorrhage and contrast enhancement after endovascular embolization of cSDH compared to conventional CT images and flat-panel CTs. This is important since our data also show that certain enhancement patterns after embolization may increase the risk for a persistent or even progressive cSDH on short- and long-term follow-up. Internal enhancement within the hematoma and the presence of a contrast medium–blood fluid–fluid level may serve as potential imaging biomarkers for unfavorable outcomes, although their clinical utility remains to be established. These findings could highlight potential variables or targets for future trials.

A conceivable reason for this finding could be that, in cases of severe leakage of contrast media into the hematoma, embolization, especially with small particles, might not be sufficient. This might be particularly true for a histopathological subtype of cSDH with hemorrhagic inflammation (26). These hematomas exhibit capillaries with a large lumen on the external side of the outer membrane and many thin vessels on the side of the hematoma cavity. This could lead to pronounced trespassing of blood, contrast media, and even small particles into the hematoma cavity, thus rendering the intervention unsuccessful. Increased exudation from these microcapillaries has been demonstrated by the administration of technetium-99 m human serum albumin and subsequent measurement of the radioactivity level in the evacuated hematoma and the peripheral blood (27). Higher exudations were discovered in hematomas with high density or mixed density, suggesting increased leakage into the hematoma. This exudation can be at least partially predicted by the radiological appearance of the hematoma on unenhanced CTs and, more reliably, on MRI and dual-energy CT (28, 29). Monitoring patients immediately after the intervention is often done by flat-panel CT due to its easy availability in the angio suite. However, our findings suggest that areas of enhancement of cSDHs can be more reliably identified using PCCT. Even if this technology is not yet widely available, dual-energy CT can be used for this purpose as well (21) and quantification of iodine and hemorrhage *in vitro* and *in vivo* is possible with both CT technologies (21, 25).

Almost all of our patients were embolized using small particles (45–150 μm, Contour polyvinyl alcohol (PVA), Boston Scientific, Natick, Massachusetts, USA) with coiling of the main trunk of the MMA. The reason for this choice of embolic material is the possibility to perform the intervention under local anesthesia, thus saving hospital resources and closely monitoring the patients during the intervention. However, incomplete occlusion with small particles cannot be sufficiently compensated for with coils of the main trunk of the MMA due to the potential mechanism of leakage into the cSDH, which might continue via meningeal collaterals from the contralateral side. Leakage might be inhibited more effectively by using liquid embolic agents than by using particles to prevent the recurrence of cSDHs, despite their downsides, such as the need for conscious sedation or intubation anesthesia (30). The three large randomized controlled trials used liquid embolics for MMA embolization (17–19). Prospective Data for particles, on the other hand, are not yet available. However, the collection of contrast media within the hematoma seems to be a potential factor promoting recurrence and may therefore be used to monitor these patients more closely in the short-term as well as long-term follow-up. Additionally, enhancement patterns could even be a potential diagnostic marker to select patients eligible for successful MMA embolization.

We demonstrated that using PCCT significantly increases the diagnostic confidence of raters, although both raters were skilled neuroradiologists with experience in both MMA embolization and PCCT. This underscores the superiority of PCCT compared to flat-panel CT and even CI. However, differences in the levels of training and experience among raters and lack of generally accepted criteria for evaluating enhancement patterns in cSDH might lead to subjective interpretations and inconsistencies among raters. A potential solution for this issue might be standardized or structured reporting as well as specific training to increase homogeneous assessment of post-interventional CTs, be it a flat-panel CT, a conventional CT or PCCT (31).

Limitations of this study are various. This is a single center retrospective observational study with limited generalizability of our findings. Both patient groups examined with flat-panel CT or PCCT might be created with considerable selection bias and were ultimately rated using both techniques, although a consensus reading was used for final assessment. Detailed information on anticoagulant and antiplatelet therapy was not consistently available, due to the focus an imaging parameter, which may have influenced outcomes but were not assessed in this study. Furthermore, the significant difference in timing between flat-panel CT and PCCT imaging introduces a potential confounder, as delayed imaging may influence the visibility of contrast enhancement patterns. Additionally, the raters were not specifically trained to report the enhancement patterns, which might have improved consistency and interrater agreement and they were not blinded to the imaging modalities. Nevertheless, in a considerable number of cases, we demonstrated that specific enhancement patterns were more frequently observed in patients who exhibited hematoma recurrence. This finding might have potential for prognostication and guiding future research. Especially the potential connection between histopathological subtypes of cSDH and their prediction with PCCT seems promising. Additionally, we demonstrated that PCCT provides greater diagnostic confidence in identifying different enhancement patterns, indicating its potential for future application in post embolization settings. Further studies should aim to confirm these findings and establish their generalizability.

## Conclusion

Our study demonstrates that PCCT significantly enhances diagnostic confidence in differentiating hyperdense hemorrhage from contrast enhancement in cSDH after MMA embolization. This distinction is clinically relevant, as specific post-embolization enhancement patterns—particularly internal enhancement and fluid–fluid levels—could x be associated with unfavorable short- and long-term outcomes.

## Data Availability

The raw data supporting the conclusions of this article will be made available by the authors, without undue reservation.

## References

[1] Wepfer JJ. Observationes anatomicae, ex cadaveribus eorum, quos sustulit apoplexia: Cum exercitatione de eius loco affecto. Schaffhusii: Waldkirch (1675).

[2] Virchow R. Das Hämatom der Dura mater. Verhandlungen der Physikalisch-Medizinischen Gesellschaft zu Würzburg. (1857) 7:134–42.

[3] Uno M. Chronic subdural hematoma-evolution of etiology and surgical treatment. Neurol Med Chir. (2023) 63:1–8. doi: 10.2176/jns-nmc.2022-0207, PMID: 36288974 PMC9894619

[4] Huang M Dai J Zhong X Wang JI Xu J Du BO. The pathogenesis of chronic subdural hematoma in the perspective of neomembrane formation and related mechanisms. Biocell. (2024) 48:889–96. doi: 10.32604/biocell.2024.050097, PMID: 40296910

[5] Shapiro M Walker M Carroll KT Levitt MR Raz E Nossek E. Neuroanatomy of cranial dural vessels: implications for subdural hematoma embolization. J NeuroInterv Surg. (2021) 13:471–7. doi: 10.1136/neurintsurg-2020-016798, PMID: 33632880

[6] Qiu S Zhuo W Sun C Su Z Yan A Shen L. Effects of atorvastatin on chronic subdural hematoma: a systematic review. Medicine. (2017) 96:e7290. doi: 10.1097/MD.0000000000007290, PMID: 28658127 PMC5500049

[7] Poulsen FR Munthe S Søe M Halle B. Perindopril and residual chronic subdural hematoma volumes six weeks after burr hole surgery: a randomized trial. Clin Neurol Neurosurg. (2014) 123:4–8. doi: 10.1016/j.clineuro.2014.05.003, PMID: 25012003

[8] Hutchinson PJ Edlmann E Bulters D Zolnourian A Holton P Suttner N. Trial of dexamethasone for chronic subdural hematoma. N Engl J Med. (2020) 383:2616–27. doi: 10.1056/NEJMoa2020473, PMID: 33326713

[9] Kim E. Embolization therapy for refractory hemorrhage in patients with chronic subdural hematomas. World Neurosurg. (2017) 101:520–7. doi: 10.1016/j.wneu.2017.02.070, PMID: 28249828

[10] Onyinzo C Berlis A Abel M Kudernatsch M Maurer CJ. Efficacy and mid-term outcome of middle meningeal artery embolization with or without burr hole evacuation for chronic subdural hematoma compared with burr hole evacuation alone. J NeuroInterv Surg. (2022) 14:297–300. doi: 10.1136/neurintsurg-2021-017450, PMID: 34187870

[11] Ku JC Dmytriw AA Essibayi MA Banihashemi MA Vranic JE Ghozy S. Embolic agent choice in middle meningeal artery embolization as primary or adjunct treatment for chronic subdural hematoma: a systematic review and Meta-analysis. AJNR Am J Neuroradiol. (2023) 44:297–302. doi: 10.3174/ajnr.A7796, PMID: 36797028 PMC10187811

[12] Sattari SA Yang W Shahbandi A Feghali J Lee RP Xu R. Middle meningeal artery embolization versus conventional Management for Patients with Chronic Subdural Hematoma: a systematic review and Meta-analysis. Neurosurgery. (2023) 92:1142–54. doi: 10.1227/neu.0000000000002365, PMID: 36929762

[13] Chen H Colasurdo M Kan PT. Middle meningeal artery embolization as standalone treatment versus combined with surgical evacuation for chronic subdural hematomas: systematic review and meta-analysis. J Neurosurg. (2024) 140:819–25. doi: 10.3171/2023.7.JNS231262, PMID: 37877965

[14] Fiehler J Bechstein M. Does every subdural hematoma patient need an embolization? Clin Neuroradiol. (2024) 34:289–91. doi: 10.1007/s00062-024-01425-z, PMID: 38753157 PMC11130060

[15] Ban SP Hwang G Byoun HS Kim T Lee SU Bang JS. Middle meningeal artery embolization for chronic subdural hematoma. Radiology. (2018) 286:992–9. doi: 10.1148/radiol.2017170053, PMID: 29019449

[16] Link TW Rapoport BI Paine SM Kamel H Knopman J. Middle meningeal artery embolization for chronic subdural hematoma: endovascular technique and radiographic findings. Interv Neuroradiol. (2018) 24:455–62. doi: 10.1177/1591019918769336, PMID: 29720020 PMC6050895

[17] Fiorella D Monteith SJ Hanel R Atchie B Boo S McTaggart RA. Embolization of the middle meningeal artery for chronic subdural hematoma. N Engl J Med. (2025) 392:855–64. doi: 10.1056/NEJMoa2409845, PMID: 39565980

[18] Liu J Ni W Zuo Q Yang H Peng Y Lin Z. Middle meningeal artery embolization for nonacute subdural hematoma. N Engl J Med. (2024) 391:1901–12. doi: 10.1056/NEJMoa2401201, PMID: 39565989

[19] Davies JM Knopman J Mokin M Hassan AE Harbaugh RE Khalessi A. Adjunctive middle meningeal artery embolization for subdural hematoma. N Engl J Med. (2024) 391:1890–900. doi: 10.1056/NEJMoa2313472, PMID: 39565988

[20] Nie W Jiang W Huang H Xu G Hu Q Zhou H. Efficacy and safety of middle meningeal artery embolization for nonacute subdural hematoma. J Neurol. (2025) 272:309. doi: 10.1007/s00415-025-13029-9, PMID: 40175600

[21] Bodanapally UK Fleiter TR Aarabi B Malhotra A Gandhi D. Dual-energy CT imaging of chronic subdural hematoma membranes: technical note. Eur Radiol. (2023) 33:797–802. doi: 10.1007/s00330-022-09064-z, PMID: 35999369

[22] Bodanapally UK Aarabi B Liang Y Khalid M Fleiter TR Gandhi D. Quantitative DECT of iodine in chronic subdural hematoma as surrogate of membrane exudation: a pilot feasibility study. J Comput Assist Tomogr. (2023) 47:951–8. doi: 10.1097/RCT.0000000000001501, PMID: 37948371

[23] Mureb MC Kondziolka D Shapiro M Raz E Nossek E Haynes J. DynaCT enhancement of subdural membranes after middle meningeal artery embolization: insights into pathophysiology. World Neurosurg. (2020) 139:e265–70. doi: 10.1016/j.wneu.2020.03.188, PMID: 32298816

[24] Maurer CJ Berlis A Stangl FJ Behrens L. In vivo discrimination of iodine and tantalum-based liquid Embolics after intracranial or spinal embolization using photon-counting detector CT. Clin Neuroradiol. (2025). doi: 10.1007/s00062-025-01502-x, PMID: 39915306 PMC12454468

[25] Risch F Berlis A Kroencke T Schwarz F Maurer CJ. Discrimination of hemorrhage and contrast Media in a Head Phantom on photon-counting detector CT data. AJNR Am J Neuroradiol. (2024) 45:183–7. doi: 10.3174/ajnr.A8093, PMID: 38164551 PMC11285985

[26] Gandhoke GS Kaif M Choi L Williamson RW Nakaji P. Histopathological features of the outer membrane of chronic subdural hematoma and correlation with clinical and radiological features. J Clin Neurosci. (2013) 20:1398–401. doi: 10.1016/j.jocn.2013.01.010, PMID: 23916760

[27] Tokmak M Iplikcioglu AC Bek S Gökduman CA Erdal M. The role of exudation in chronic subdural hematomas. J Neurosurg. (2007) 107:290–5. doi: 10.3171/JNS-07/08/0290 17695382

[28] Chen H Colasurdo M Malhotra A Gandhi D Bodanapally UK. Advances in chronic subdural hematoma and membrane imaging. Front Neurol. (2024) 15:1366238. doi: 10.3389/fneur.2024.1366238, PMID: 38725642 PMC11079242

[29] Tanikawa M Mase M Yamada K Yamashita N Matsumoto T Banno T. Surgical treatment of chronic subdural hematoma based on intrahematomal membrane structure on MRI. Acta Neurochir. (2001) 143:613–9. doi: 10.1007/s007010170067, PMID: 11534679

[30] Salem MM Kuybu O Nguyen Hoang A Baig AA Khorasanizadeh M Baker C. Middle meningeal artery embolization for chronic subdural hematoma: predictors of clinical and radiographic failure from 636 Embolizations. Radiology. (2023) 307:e222045. doi: 10.1148/radiol.222045, PMID: 37070990 PMC10323293

[31] Ganeshan D PAT D Probyn L Lenchik L Mc Arthur TA Retrouvey M. Structured reporting in radiology. Acad Radiol. (2018) 25:66–73. doi: 10.1016/j.acra.2017.08.005, PMID: 29030284

